# Designing biomimetic two-dimensional channels for uranium separation from seawater†

**DOI:** 10.1039/d4sc02801e

**Published:** 2024-06-10

**Authors:** Wenbin Liang, Xin Zhang, Liqin Wang, Chuanxi Wen, Longlong Tian, Zhan Li, Ximeng Chen, Wangsuo Wu

**Affiliations:** a MOE Frontiers Science Center for Rare Isotopes, Lanzhou University Lanzhou 730000 China tianll12@lzu.edu.cn liz@lzu.edu.cn; b School of Nuclear Science and Technology, Lanzhou University Lanzhou 730000 China; c School of Chemistry and Chemical Engineering, Qinghai Nationalities University Xining 810007 China

## Abstract

Efficient separation of uranium from seawater stands as a pivotal challenge. This study unveils an approach focusing on the ingenious design of biomimetic two-dimensional (2D) membranes tailored explicitly for this purpose. Leveraging the unique interplay of DNA strands housing U aptamers, pH-responsive i-motifs, and poly A_(10)_ segments ingeniously embedded within graphene oxide membranes, a distinctive biomimetic 2D channel is engineered. The strategic integration of these bio-inspired elements enables dynamic adjustment of interlayer spacing, augmenting both the permeability of the membrane and the selectivity of the aptamer for uranyl ions. During the separation process, the encounter between uranyl ions and the enhanced aptamer within the interlayers initiates a crucial interaction, triggering a specific concentration polarization mechanism. This mechanism stands as the cornerstone for achieving a highly selective separation of uranyl ions from the vast and complex matrix of seawater. The membrane exhibits excellent performance in real seawater, with a rejection rate of uranyl ions of ≈100% and sustained selectivity of uranyl ions over ten cycles. Importantly, the selectivity of uranium and vanadium can reach 14.66. The significance of this research lies not only in the effective separation of uranyl ions but also in showcasing the broader applicability of 2D membrane design in chemical engineering.

## Abstract 2

Design and construction of a biomimetic two-dimensional channel using biomolecules and efficient extraction of uranyl ions from seawater.

## Graphical abstract

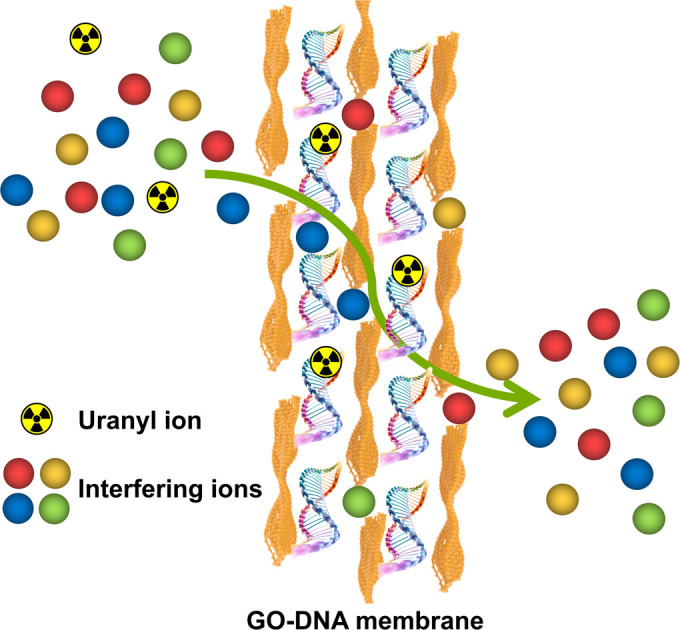

## Introduction

Nuclear energy represents a significant avenue for meeting the escalating demands of future energy requirements.^1–3^ Uranium remains the predominant fuel source for most nuclear power plants.^4,5^ Conventionally, uranium extraction has been predominantly reliant on underground deposits.^6,7^ However, the limited reserves of such deposits necessitate the exploration of alternative uranium sources. Seawater presents a viable option as it contains minute concentrations of uranium (approximately 3.3 ppb).^8,9^ Given the vast volume of seawater, the total uranium resource potential is staggering, estimated to be approximately 4.5 billion tons, surpassing land-based proven uranium reserves by several orders of magnitude.^6^ Harnessing uranium resources from the ocean in an efficient manner holds substantial promise for the sustainable advancement of nuclear power. Currently, adsorption is the mainstream method for extracting uranium from seawater, but there is no ideal adsorbent, which limits further development.^4,10–12^ In contrast, membrane separation processes have been widely used in various fields due to their phase-change-free and additive-free separation capabilities.^13–16^ However, developing a highly selective and permeable membrane for uranium extraction from seawater presents a significant challenge.

A two-dimensional (2D) lamellar membrane represents a separation membrane constructed through the sequential stacking of 2D nanosheets.^14^ The interlayer channels, emerging from the stacking arrangement between these nanosheets, facilitate the swift and discerning transport of small molecules.^17^ Consequently, the 2D lamellar membrane stands as an exemplary medium for the separation of small molecules.^13^ Graphene oxide (GO), a prominent derivative of graphene, assumes a crucial role as a 2D constituent within these lamellar membranes.^18^ The GO membrane, owing to its commendable mass transfer capabilities, has garnered considerable attention from researchers in recent years and undergone comprehensive development. Notably, it has made significant strides in diverse fields such as seawater desalination, metal ion separation, gas separation, radioactive material separation, and isotope separation, establishing itself as a pivotal element in the realm of membrane-based separation technologies.^19–29^

Despite the sustained advancements in the realm of small molecule separation achieved by 2D lamellar membranes, a universally accepted theoretical framework for elucidating the separation mechanisms remains elusive.^15^ Predominantly, extant theories underscore the significance of the size screening effect in facilitating the selective separation of 2D lamellar membranes.^30^ It is widely recognized that GO, being a pliable material, brings a layer of complexity to size screening.^31–34^ Recent investigations reveal that metal ions possess the capability to permeate the interlayer space of the GO membrane, thereby exerting influence over its interlayer spacing.^19^ In scenarios involving mixed ion systems, the determination of the GO membrane's interlayer spacing hinges upon the initial entry of metal ions into the interlayer space.^35^ However, the flexible nature of materials such as GO implies that the size screening effect alone fails to comprehensively account for all observed separation phenomena. Consequently, it is imperative to posit the existence of an alternative separation mechanism beyond the confines of the size screening effect.

Aptamers, DNA or RNA fragments capable of specific molecular recognition, were initially discovered in 1994 and have since found extensive utility in trace metal ion detection.^36,37^ Lu *et al.* employed a combinatorial biology approach to successfully isolate the uranyl ion recognition aptamer (U Aptamer), notable because of its high specificity towards uranyl ions (UO_2_^2+^) and its applicability in detecting minute quantities of this species.^38^ Recently, Wang *et al.* harnessed the U Aptamer in uranium extraction from seawater, yielding promising outcomes.^4^ The i-motif, characterized by a cytosine-rich DNA sequence, exhibits pH-dependent secondary structure dynamics.^39–42^ Specifically, its secondary structure remains negligible under alkaline conditions, whereas it adopts a quadruplex configuration under acidic environments, with increased folding propensity correlating with heightened acidity. Moreover, DNA molecules exhibit adsorption onto GO surfaces *via* π–π interactions primarily involving nitrogenous bases. Notably, purine bases (such as adenine and guanine) bind more strongly than pyrimidine bases (such as cytosine and thymine), as evidenced by research findings.^43^

**Scheme 1:**
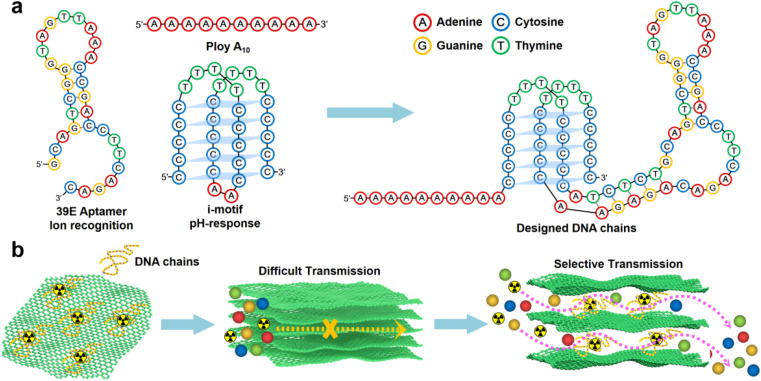
Schematic illustration of a 2D membrane with ion recognition channels. (a) Schematic representation of DNA fragments composed of poly A_(10)_, U Aptamer and i-motif fragments. (b) Construction strategy of the 2D separation membrane.

In this study, a designed DNA chain comprising poly A_(10)_, i-motif, and U Aptamer was employed (Scheme 1a).^44^ The DNA chain was strategically inserted between the layers of GO. The poly A_(10)_ chains demonstrated an affinity to the GO surface through π–π interactions. The inclusion of an ion-binding aptamer enhanced membrane selectivity, while the i-motif played a pivotal role in dynamically adjusting the spacing between GO layers based on the system pH, thereby regulating permeability (Scheme 1b). Following the introduction of the DNA construct, a notable inhibition in the transmembrane transport of UO_2_^2+^ was observed. Intriguingly, the transmembrane transport of VO^2+^ remained unaffected. Under optimal conditions, the U/V separation factor (SF) reached a noteworthy value of 14.66. A novel separation mechanism is proposed to elucidate this distinctive phenomenon, providing valuable insights into the separation mechanisms governing 2D lamellar membranes. The outcomes of this research not only bear significance for the tailoring of 2D lamellar membranes but also extend their impact to the broader realm of advancing separation technologies within the chemical field.

## Results and discussion

### Interaction and stability between GO and DNA

**Fig. 1:**
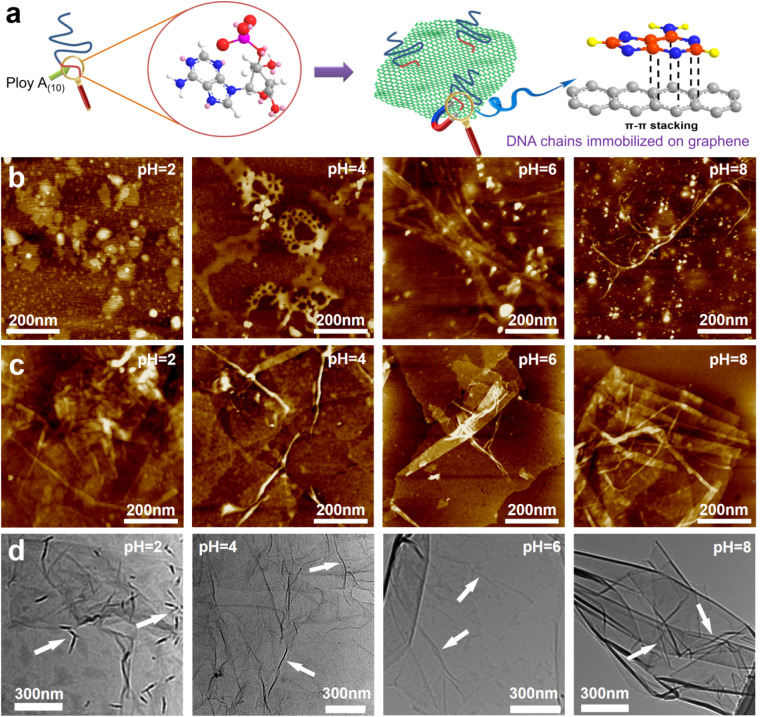
Interaction between GO and DNA. (a) Schematic diagram of π–π stacking between DNA bases and GO. (b) AFM images of DNA at pH = 2, 4, 6 and 8. The white particles in the image are crystals of the buffer solution used to adjust the pH. AFM image (c) and TEM image (d) of the mixture of GO and DNA at pH = 2, 4, 6 and 8.

GO was synthesized using a modified Hummers' method. The DNA chains used in the experiment were purchased directly from Suzhou Biosyntech Co., Ltd. The sequences of the DNA molecules are shown in Section 2 of the ESI.† The adsorption efficacy of GO towards the engineered DNA was evaluated to substantiate the efficacy of immobilizing DNA molecules within the interlayer spaces of GO *via* π–π interactions. The results revealed near-complete adsorption of DNA onto GO (with the post-adsorption DNA concentration falling below the detection threshold of the instrument, ESI,† Fig. S1a). The selectivity of the DNA sequences towards UO_2_^2+^ was assessed in spiked simulated seawater. The findings demonstrate the selective recognition and adsorption capability of the DNA towards UO_2_^2+^ (ESI,† Fig. S1b). These results collectively affirm the efficacy of the separation strategy employed. Subsequently, GO–DNA membranes were prepared through the negative pressure suction filtration method. DNA becomes adsorbed onto the GO surface *via* π–π stacking interactions between DNA bases and GO, as illustrated in Fig. 1a. In this investigation, poly A_(10)_ fragments were introduced into the DNA strands to augment the π–π stacking interaction.^45–47^ The absence of UV absorption at a wavelength of 260 nm in the filtrate during membrane preparation (ESI,† Fig. S3) indicates the complete immobilization of all DNA strands within the membrane.

The morphology of DNA molecules at different pH levels (pH = 2, 4, 6, and 8) was characterized using atomic force microscopy (AFM). At pH = 2, the phosphodiester bond of the DNA molecule undergoes hydrolysis, resulting in shorter fragments and an irregular AFM image (Fig. 1b). Mass spectrometry results confirm the hydrolysis of DNA molecules (ESI† Fig. S4). Chain-like DNA molecules are observable at other pH values (pH = 4, 6, and 8). Furthermore, a mixture of GO and DNA was analyzed using AFM and transmission electron microscopy (TEM). AFM images reveal a similar phenomenon to DNA on the GO surface (Fig. 1c). Chain-like structures were observed on the GO surface at mild pH (pH = 4, 6, and 8), while irregularities were observed at pH = 2. AFM images also confirm the distribution of DNA molecules between the two layers of GO, signifying the successful insertion of DNA molecules between the GO layers. TEM images further support this observation (Fig. 1d). To eliminate the interference caused by GO folds, TEM images of GO were employed for comparative analysis (ESI,† Fig. S5). Notably, GO folds exhibit broader and longer features, coupled with a comparatively lower density of surface wrinkles at equivalent magnifications compared to the blend. This validation substantiates the identity of the numerous chain structures observed in the TEM images of GO and DNA mixtures as unequivocally DNA molecules. It is noteworthy that the distribution of DNA on the GO surface is evident in both AFM and TEM images, irrespective of whether it is an intact DNA molecule or a hydrolyzed fragment. This underscores the effectiveness of the π–π interaction between GO and DNA in anchoring DNA molecules to the surface of the GO nanosheets. Moreover, the successful insertion of DNA molecules between the GO layers post-membrane preparation is highlighted. At the same time, this observation shows that the GO–DNA membrane cannot be applied to strongly acidic systems. However, the pH value of seawater is usually in the range of 7 to 8, and the GO–DNA membrane is stable under this condition. Therefore, it is feasible to apply the GO–DNA membrane to uranium extraction from seawater.

To assess the stability of the GO–DNA complex, the GO–DNA membrane was immersed in aqueous solutions with varying pH values (pH = 2, 4, 6, and 8) for different durations. The resulting solutions were subjected to analysis using UV spectroscopy and gel electrophoresis. Remarkably, no DNA bands were observed even after 7 days of immersion (ESI,† Fig. S6), and there was no discernible UV absorption at 260 nm (ESI,† Fig. S7). This observation underscores the efficacy of the π–π interaction between DNA bases and GO in securely anchoring DNA molecules between the GO layers. Importantly, this stability persists even when the DNA molecules undergo hydrolysis, preventing their leaching from the GO layers.

### Regulation of GO layer spacing and transmittance by DNA

**Fig. 2:**
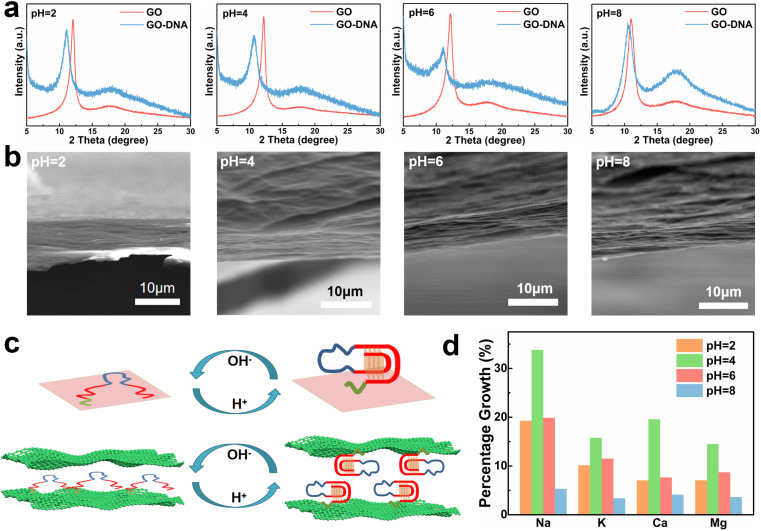
GO–DNA membrane interlayer spacing and pH dependence of ionic transmittance. (a) XRD characterization results of GO–DNA membranes at pH = 2, 4, 6 and 8. (b) SEM images of GO–DNA membrane sections at pH = 2, 4, 6 and 8. (c) Structure–activity relationship between the pH dependence of the i-motif structure and the interlayer space of GO–DNA membranes. (d) The percentage growth rate of the ionic transmittance of the GO–DNA membrane compared with the GO membrane at pH = 2, 4, 6 and 8.

GO and GO–DNA membranes were immersed in solutions with different pH values (pH = 2, 4, 6, and 8) for 12 hours, dried and then analyzed using X-ray diffraction (XRD, Fig. 2a). Upon the incorporation of DNA, the (001) peak (∼12°) of GO shifted to a lower degree, indicating an increase in the interlayer spacing of the GO membrane. The extent of the left shift of the (001) peak varied with pH. In comparison with the GO membrane, the 2*θ* degree of the GO–DNA membrane shifted left by 1°, 1.45°, 1.1°, and 0.38° at pH = 2, 4, 6, and 8, respectively (ESI,† Fig. S8). This pH-dependent change in interlayer spacing is attributed to the influence of the pH-dependent i-motif structure. Under alkaline conditions, where the i-motif secondary structure is negligible, the expansion of the interlayer spacing is primarily attributed to the size of the DNA molecule itself. Conversely, under acidic conditions, the formation of an i-motif tetrad structure results in a significant increase in the interlayer spacing of GO (Fig. 2c). And as the pH decreases, this folding process intensifies, leading to slight differences in the interlayer spacing of GO–DNA membranes under acidic conditions. At pH = 2, DNA molecules undergo hydrolysis, and mass spectrometry data directly verify the hydrolysis of DNA molecules (ESI,† Fig. S4). Immersion experiments confirm that hydrolyzed fragments persist within the interlayer space (ESI,† Fig. S6 and S7). The widening of the interlayer spacing of GO is a result of the accumulation of these fragments post-hydrolysis, a process dictated by the inherent properties of the DNA molecule itself.

Changes in the interlayer spacing of the GO membrane directly affect its ionic transmittance. Typical monovalent ions (Na^+^ and K^+^) and divalent ions (Ca^2+^ and Mg^2+^) were used to evaluate the ionic transmittance of the GO–DNA membrane in a custom-made permeable membrane separation device (ESI,† Fig. S1a). The ionic transmittance data for the GO membrane and GO–DNA membrane under varying pH conditions are presented in ESI† Fig. S9. The growth rate of ionic transmittance was calculated to evaluate the change in ionic transmittance after the introduction of DNA molecules (Fig. 2d). Specifically, at pH = 2, the ionic transmittance of Na^+^ increased by 19.26%, 33.78%, 19.87%, and 5.32% respectively at pH = 2, 4, 6, and 8. Similarly, the ionic transmittance of K^+^ showed increases of 10.15%, 15.76%, 11.49%, and 3.35% at the same pH values. The ionic transmittance trends for Ca^2+^ and Mg^2+^ exhibited similar patterns. The influence of pH on the interlayer spacing was highly consistent with its impact on ionic transmittance. These results suggest that DNA molecules can dynamically adjust the interlayer spacing of the GO membrane in response to the system's pH, thereby modulating its ionic transmittance.

The GO–DNA membranes were subjected to characterization under different pH values (pH = 2, 4, 6, 8) using scanning electron microscopy (SEM). The cross-section of the GO–DNA membrane reveals a distinctive “book-like” layered structure (Fig. 2b), consistent with the typical structure of GO membranes. Raman spectroscopy was employed to investigate defects in GO membranes both before and after DNA incorporation and before and after separation (ESI,† Fig. S10). No significant alterations in GO defects were observed under these conditions. Fourier transform infrared spectroscopy (FT-IR, ESI,† Fig. S11) was utilized to analyze the chemical composition of both GO and GO–DNA membranes. Compared with the GO membrane, the peaks at 1240 cm^−1^ and 1070 cm^−1^ of the GO–DNA membrane are significantly enhanced, which are attributed to P=O and P–O in the DNA molecules respectively, proving the successful introduction of DNA.

### Separation performance of the GO–DNA membrane

**Fig. 3:**
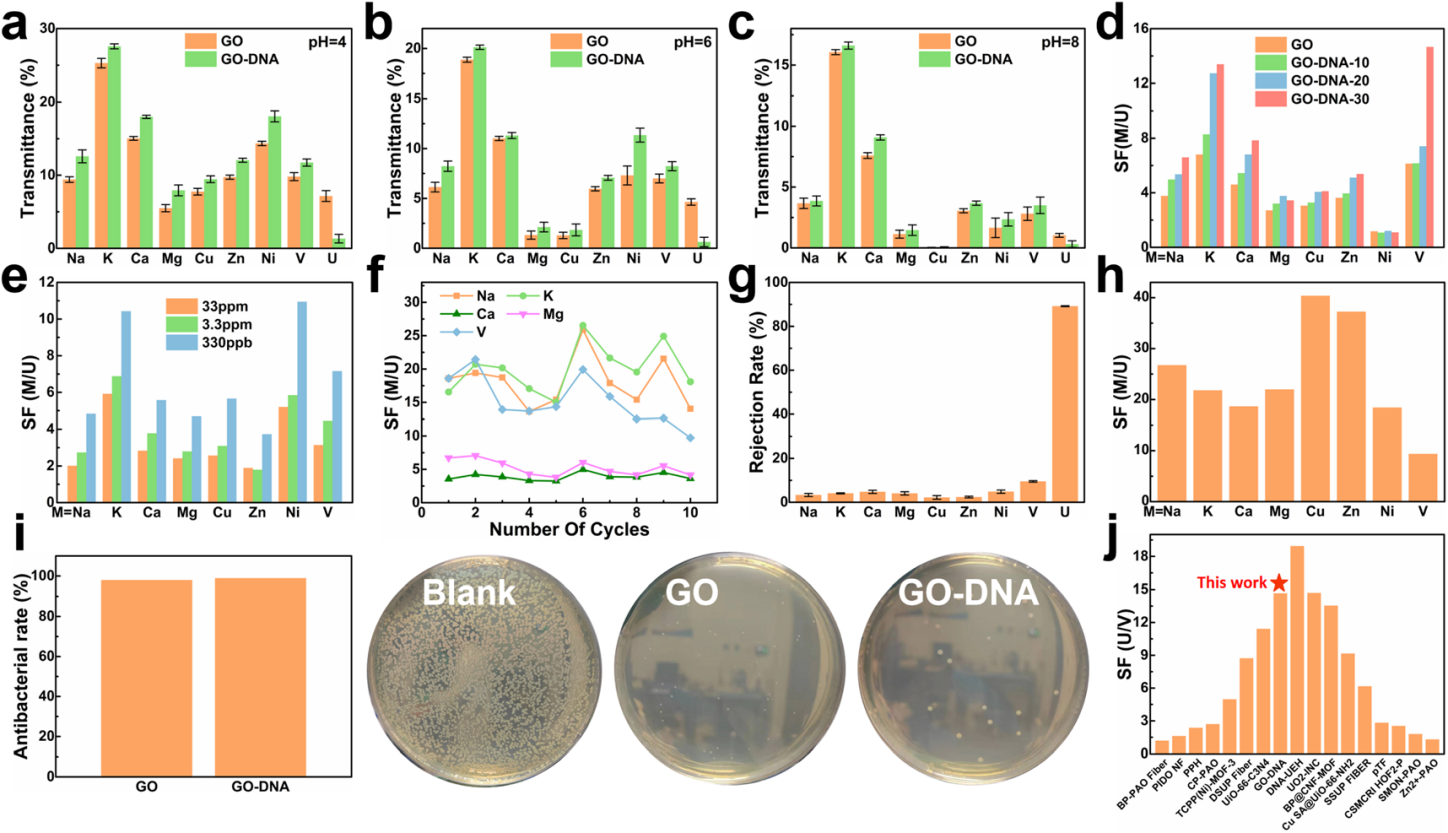
Separation performance test of the GO–DNA membrane. Transmittance of the GO membrane and GO–DNA membrane in spiked simulated seawater at pH = 4 (a), pH = 6 (b) and pH = 8 (c). Separation factors after 12 hours of separation experiment of the GO–DNA membrane with different amounts of DNA added (d) and different uranium spiked concentrations (e). (f) The separation factor of the GO–DNA membrane that was recycled ten times in spiked real seawater without any treatment. (g) Ion rejection rate and (h) separation factor of the GO–DNA membrane in pressure filtration experiments at a pressure of 0.08 Mpa. (i) Antibacterial ability of the GO membrane and GO–NDA membrane. (j) Comparison of uranium and vanadium separation performance.^4,7,48–61^

To assess the selectivity of the GO–DNA membrane, its performance in separating uranium was examined in spiked simulated seawater. For convenience, the concentrations of Cu^2+^, Ni^2+^, Zn^2+^, and UO_2_^2+^ were increased by a thousand-fold (the concentrations of all ions are given in ESI† Table S1). The impact of pH on the separation efficiency of the GO–DNA membrane was investigated at pH values of 4, 6, and 8. Ionic transmittance after a 12-hour separation experiment is presented in Fig. 3a–c. Remarkably, GO–DNA demonstrates exceptional rejection of UO_2_^2+^ at all tested pH levels. This underscores the outstanding separation performance of GO–DNA membranes under pH conditions conducive to the stability of DNA molecules. In certain instances, an observation of higher transmittance for divalent ions compared to monovalent ions has been documented. This occurrence can be ascribed to the disparate initial concentrations of metal ions. Specifically, in spiked simulated seawater, the concentration of monovalent ions greatly surpasses that of divalent ions. While the percentage transmission of monovalent ions may seem comparatively lower than that of divalent ions, their absolute transmission capacity is substantially greater.

The influence of the added amount of DNA on the GO–DNA separation effect was further probed. The separation performance of GO–DNA membranes with different amounts of DNA added was tested in spiked simulated seawater (labeled GO–DNA-*x*, where *x* represents the amount of DNA added to the separation membrane in nmol). Ionic transmittance after the 12-hour separation experiment is depicted in Fig. S12a,† and the SF is shown in Fig. 3d. As the amount of DNA added increases, the transmittance of UO_2_^2+^ gradually decreases, while the transmittance of other ions exhibits minor changes. Consequently, the SF of coexisting ions and UO_2_^2+^ increases with the augmented DNA content, affirming that the introduction of DNA enhances the UO_2_^2+^ selectivity of the GO membrane.

Given the extremely low UO_2_^2+^ content in natural seawater (only 3.3 ppb), testing the application prospects of the GO–DNA membrane in seawater necessitated a reduction in UO_2_^2+^ concentration in spiked seawater. Its separation performance was evaluated using various spiked concentrations of uranyl ions (33 ppm, 3.3 ppm, and 330 ppb, respectively). The concentrations of the remaining ions were the same as those of the spiked simulated seawater, as shown in ESI† Table S1. Ionic transmittance after a 12-hour separation experiment is illustrated in Fig. S12b,† and the SF is shown in Fig. 3e. The transmittance of UO_2_^2+^ decreases as its concentration decreases, while the transmittance of other ions remains relatively stable. This signifies that GO–DNA maintains excellent separation ability even at lower UO_2_^2+^ concentrations, indicating promising prospects for seawater applications.

Cycling stability, a crucial parameter for industrial membrane applications, was evaluated in spiked real seawater without membrane regeneration. The concentrations of all ions were the same as those of spiked simulated seawater, as shown in ESI† Table S1. Even after undergoing ten cycles, the transmittance of all ions exhibited fluctuations within a specific range, with the rate of change in ionic transmittance remaining between −40% and 40% (ESI,† Fig. S13a and b). Additionally, in the cycling stability experiment, the rejection rate of UO_2_^2+^ by the GO–DNA membrane remained consistent, while the SF between UO_2_^2+^ and coexisting ions fluctuated within an established range (Fig. 3f). The concentration of UO_2_^2+^ in the source solution was tested to explore the UO_2_^2+^ capturing ability of the GO–DNA membrane (ESI,† Fig. S13c). After ten cycles, the UO_2_^2+^ concentration showed only a weak decrease. Considering that a small amount of UO_2_^2+^ was detected in the driving solution (ESI,† Fig. S13a), the GO–DNA membrane captures only trace amounts of UO_2_^2+^. The GO–DNA membrane mainly blocks the transmembrane transport of UO_2_^2+^, thereby achieving the separation of UO_2_^2+^. These findings demonstrate the excellent cycling stability of the GO–DNA membrane, highlighting its potential for practical industrial applications. At the same time, the excellent stability of the GO–DNA membrane provides additional evidence that the DNA sequence is stable during the separation process.

Pressure filtration experiments were conducted to comprehensively assess the efficacy of the GO–DNA membrane. Initially, the water permeability of the GO–DNA membrane was examined under varying concentrations of DNA. The findings demonstrated a positive correlation between the amount of DNA incorporated and the water permeability of the GO–DNA membrane, as illustrated in Fig. S14 of the ESI.† Subsequently, the ion separation capability of the GO–DNA membrane was evaluated using spiked real seawater. The concentrations of all ions were the same as those of spiked simulated seawater, as shown in ESI† Table S1. Fig. 3g presents the ion rejection rates, while Fig. 3h illustrates the SF for coexisting ions, specifically focusing on UO_2_^2+^. The outcomes revealed the outstanding uranyl ion rejection capability of the GO–DNA membrane along with its notably low rejection of coexisting ions. These results underscore the superior separation performance of the GO–DNA membrane in pressure filtration experiments.

**Fig. 4:**
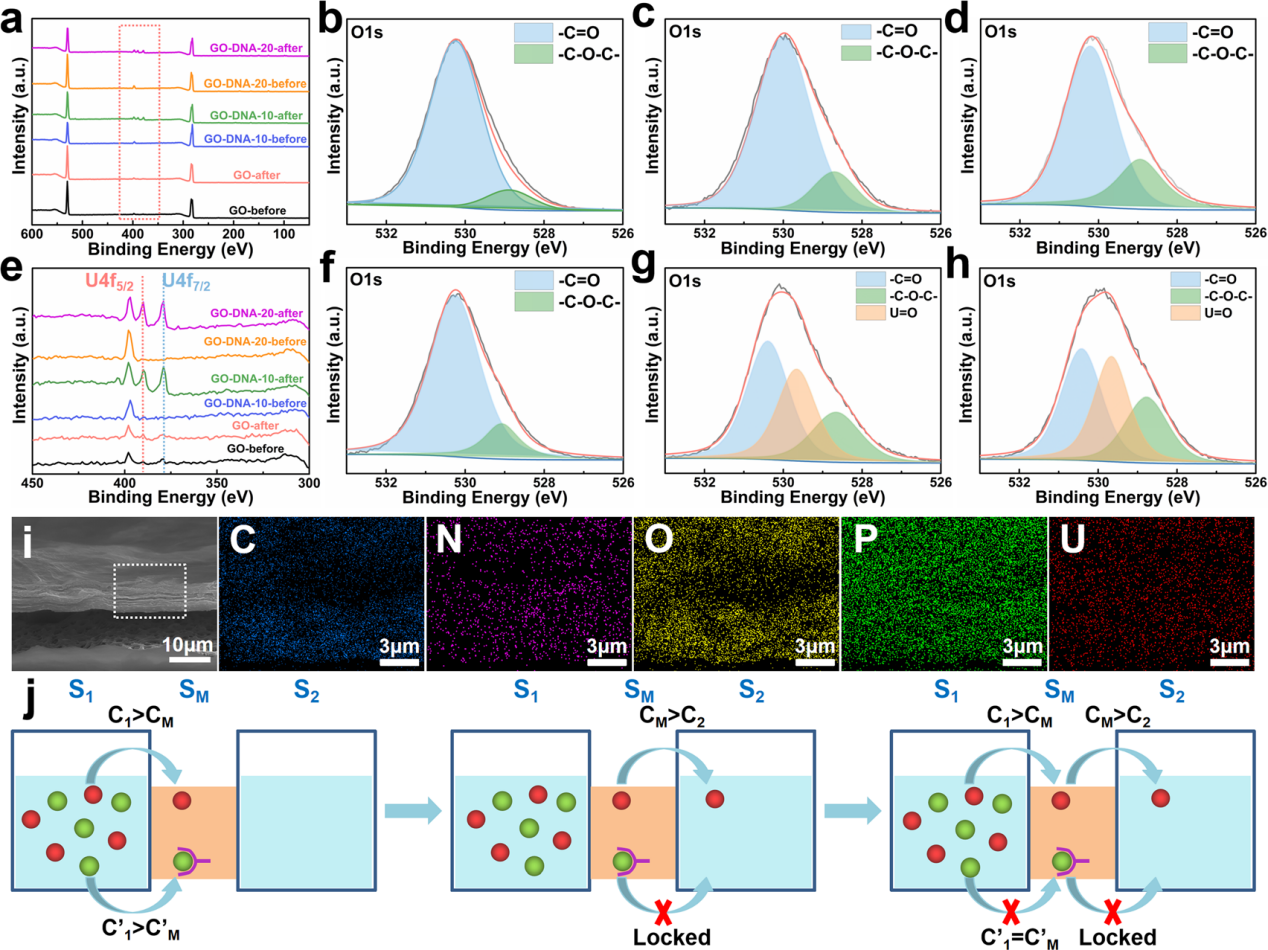
Separation mechanism. XPS analysis results of the GO membrane, GO–DNA-10 membrane and GO–DNA-20 membrane before and after the separation experiment (a) and (e) the zoomed-in results of the red box part in (a). High-resolution XPS analysis results of oxygen element in GO membranes ((b) before separation and (f) after separation), GO–DNA-10 membranes ((c) before separation and (g) after separation) and GO–DNA-20 membranes ((d) before separation and (h) after separation). (i) EDS mapping of the GO–DNA membrane after the separation. (j) Schematic diagram of ion transport across a two-dimensional membrane.

Anti-biofouling activity plays a pivotal role in the practical utilization of GO–DNA membranes. The antibacterial efficacy of the GO–DNA membrane was evaluated as per the methodologies outlined in ESI 1.12.† Both the GO membrane and the GO–DNA membrane exhibited remarkable antibacterial properties, demonstrating an antibacterial rate exceeding 98% (Fig. 4g and ESI† Table S2). This underscores the outstanding anti-biofouling performance of the GO–DNA membrane, primarily attributed to the presence of GO,^62^ with the incorporation of DNA not altering this characteristic. These findings underscore the vast potential applications of the GO–DNA membrane. The uranium-to-vanadium (U/V) separation capability stands as a crucial metric for assessing the effectiveness of membranes in uranium extraction from seawater. In our study, we compared the U/V separation capabilities of the GO–DNA membrane with those reported in recent literature on uranium extraction from seawater (Fig. 3j and ESI† Table S3).^4,7,48–61^ The comparative analysis reveals that the GO–DNA membrane exhibits outstanding U/V separation ability, underscoring its potential as an efficient separation membrane for uranium extraction from seawater.

### Separation mechanism

The GO–DNA membrane underwent rigorous scrutiny *via* X-ray photoelectron spectroscopy (XPS) both pre- and post-separation experimentation. Relative to the unmodified GO membrane, discernible features emerged in the XPS spectrum of the separated GO–DNA membrane at 390.00 eV and 379.00 eV, corresponding to U 4f_5/2_ and U 4f_7/2_, respectively (Fig. 4a and e). Notably, the presence of uranium in the separated GO–DNA membrane was confirmed by energy-dispersive X-ray spectroscopy (EDS) mapping (Fig. 4i). This evidentiary manifestation unequivocally affirms the selective entrapment of UO_2_^2+^ within the interlayer interstice.

High-resolution XPS analysis of the oxygen fraction before and after separation revealed a discernible peak at 529.65 eV in the GO–DNA membrane after separation, which was attributed to the axial oxygen of UO_2_^2+^ (Fig. 4b–d, f–h). In addition, in the GO–DNA membrane, the peak corresponding to the carbonyl oxygen shifted from 530.00 eV to 530.40 eV, a phenomenon not observed in the GO membrane. Further examination of the high-resolution XPS spectrum of uranium in the GO–DNA membrane after separation revealed three distinct peaks at 389.96 eV, 381.45 eV, and 379.10 eV (ESI,† Fig. S18). Importantly, these uranium peaks were absent in the GO–DNA membrane before separation. Subsequent to separation from the GO membrane, similar peaks at 389.96 eV and 379.10 eV were detectable, albeit with significantly reduced intensity, possibly due to residual UO_2_^2+^. Meanwhile, high-resolution XPS analysis of other elemental components demonstrated minimal transformation before and after separation (ESI,† Fig. S15–S17). These findings collectively suggest that the U Aptamer binds to UO_2_^2+^ through oxygen coordination, consistent with existing literature reports.

The separation mechanism of the GO–DNA membrane is obviously different from the traditional size screening effect. A novel separation mechanism is proposed that emphasizes the role of selective concentration polarization induced within 2D lamellar membranes. Conventionally, the prevailing permeation theories treat the permeate fluid (S_1_) and driving fluid (S_2_) as discrete systems, with ion transport being contingent upon concentration gradients between them. However, the interlayer configuration of 2D lamellar membranes, characterized by an “S” shaped channel, necessitates a reevaluation of this conventional framework. In order to more precisely elucidate the intrinsic mass transfer process of 2D lamellar membranes, a completely new separation mechanism was proposed. It posits the two-dimensional separation membrane (S_M_) as an autonomous entity, beyond the conventional dichotomy of S_1_ and S_2_. The ion transport trajectory in the interlayer space assumes an “S” shape, wherein ions temporarily reside within the 2D lamellar membrane. The delineation of ion transport across 2D lamellar membranes is thus articulated through two discernible stages (Fig. 4j). The first phase involves ion diffusion from the S_1_ system into the S_M_ system, driven by concentration gradients. Given that the concentration in the S_1_ system (*C*_1_) surpasses that in the S_M_ system (*C*_M_), ions traverse from the former to the latter. Subsequently, as ions populate the S_M_ system, a situation arises where *C*_M_ exceeds the concentration in the S_2_ system (*C*_2_). This initiates the second stage, wherein ions diffuse from the S_M_ system to the S_2_ system. Collectively, these stages comprise the ion transit process across the 2D lamellar membrane. This conceptualization is further nuanced in the case of target ions, typified by active sites embedded in the interlayer space. For target ions, active sites induce a nuanced scenario. In the first stage, target ions diffuse from the S_1_ system into the S_M_ system due to concentration gradients. Upon entry into the S_M_ system, active sites recognize and immobilize the ions within the interlayer space. Despite a persisting concentration difference between the S_M_ system and the S_2_ system 
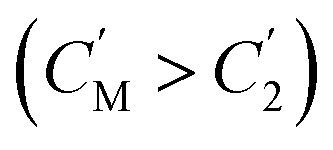
, the immobilizing effect of active sites precludes the diffusion of target ions into the S_2_ system. As target ions continue infiltrating the S_M_ system, 
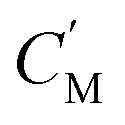
 approaches 
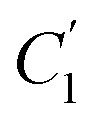
, culminating in a scenario where the concentration difference dissipates, halting the transmembrane ion transport.

In this study, UO_2_^2+^ initially permeated the S_M_ system from the S_1_ system. Subsequently, the addition of the 39E aptamer facilitated the selective recognition and binding of UO_2_^2+^, thereby immobilizing UO_2_^2+^ within the S_M_ system and preventing its translocation to the S_2_ system. Equilibrium was achieved when the concentration of UO_2_^2+^ in the S_M_ system equaled that in the S_1_ system, halting further diffusion from the S_1_ system to the S_M_ system. The transmembrane transport of UO_2_^2+^ was effectively impeded by this mechanism. Notably, the diffusion of coexisting metal ions remained unhindered, facilitating their separation from UO_2_^2+^. This selective binding action of the 39E aptamer resulted in the entrapment of a portion of UO_2_^2+^ in the S_M_ system, as evidenced by the presence of uranium in the XPS spectrum of the GO–DNA membrane. Conversely, no coexisting ions were detected in the XPS spectrum, corroborating our hypothesis. Specifically, coexisting ions will quickly diffuse into the S_2_ system after entering the S_M_ system, resulting in only very few coexisting ions existing in the GO interlayer space (S_M_ system). Their concentration remained below the detectable range of XPS analysis.

## Conclusions

In summary, the integration of DNA chains, including poly A_(10)_, U Aptamer, and pH-responsive i-motif, into GO layers resulted in sophisticated DNA-intercalated GO membranes. Poly A_(10)_ facilitated π–π interactions, binding DNA to GO, while the pH-responsive i-motif dynamically adjusted interlayer spacing. Simultaneously, the U Aptamer enhanced GO membranes' efficacy in obstructing UO_2_^2+^ transport, boosting separation efficiency. A novel 2D lamellar membrane permeability separation mechanism was proposed, redefining the 2D lamellar membranes as an independent system. This conceptual framework offers insights into enhanced separation effects, opening avenues for tailored 2D lamellar membranes. These modifications advance the understanding of 2D lamellar membrane mechanisms, paving the way for custom separation technologies. Improved separation efficiency and the proposed model highlight the potential of GO membranes for diverse applications. Future research can focus on optimizing fabrication processes, scalability, and exploring the broader applicability of this innovative separation technology.

## Data availability

The data that support the findings of this study are available on request from the corresponding author. The data are not publicly available due to privacy or ethical restrictions.

## Author contributions

W. L., X. Z., L. T., and Z. L. conceived the project. L. T. and Z. L. supervised the project. W. L., X. Z., L. W. and C. W. performed the experiments and characterization. W. L., X. Z., L. T., Z. L., X. C., and W. W. co-wrote the manuscript. All authors discussed the results and commented on the manuscript.

## Conflicts of interest

There are no conflicts to declare.

## Supplementary Material

SC-015-D4SC02801E-s001

## References

[cit1] Wang C., Helal A. S., Wang Z., Zhou J., Yao X., Shi Z., Ren Y., Lee J., Chang J. K., Fugetsu B., Li J. (2021). Adv. Mater..

[cit2] Gralla F., Abson D. J., Møller A. P., Lang D. J., Wehrden H. (2017). Renewable Sustainable Energy Rev..

[cit3] Parsons J., Buongiorno J., Corradini M., Petti D. (2019). A fresh look at nuclear energy. Science.

[cit4] Yuan Y., Liu T., Xiao J., Yu Q., Feng L., Niu B., Feng S., Zhang J., Wang N. (2020). Nat. Commun..

[cit5] Wiechert A. I., Yiacoumi S., Tsouris C. (2022). Nat. Sustain..

[cit6] Tsouris C. (2017). Nat. Energy.

[cit7] Yuan Y., Yu Q., Wen J., Li C., Guo Z., Wang X., Wang N. (2019). Angew. Chem., Int. Ed..

[cit8] Yang H., Liu X., Hao M., Xie Y., Wang X., Tian H., Waterhouse G. I. N., Kruger P. E., Telfer S. G., Ma S. (2021). Adv. Mater..

[cit9] Liu X., Xie Y., Hao M., Chen Z., Yang H., Waterhouse G. I. N., Ma S., Wang X. (2022). Adv. Sci..

[cit10] Liu T., Zhang R. Q., Chen M. W., Liu Y. J., Xie Z. J., Tang S., Yuan Y. H., Wang N. (2022). Adv. Funct. Mater..

[cit11] Wu Y., Xie Y., Liu X., Li Y., Wang J., Chen Z., Yang H., Hu B., Shen C., Tang Z., Huang Q., Wang X. (2023). Coord. Chem. Rev..

[cit12] Jian J., Kang H., Yu D., Qiao X., Liu Y., Li Y., Qin W., Wu X. (2023). Small.

[cit13] Razmjou A., Asadnia M., Hosseini E., Habibnejad K. A., Chen V. (2019). Nat. Commun..

[cit14] Liu G., Jin W., Xu N. (2016). Angew. Chem., Int. Ed..

[cit15] Liu Y., Coppens M. O., Jiang Z. (2021). Chem. Soc. Rev..

[cit16] Liu P., Hou J., Zhang Y., Li L., Lu X., Tang Z. (2020). Inorg. Chem. Front..

[cit17] Xu R., Kang Y., Zhang W., Pan B., Zhang X. (2023). Nat. Commun..

[cit18] Liang J., Liu T., Li Y., Liang W., Zhang X., Qian L., Li Z., Chen X. (2022). Cell. Rep. Phys. Sci..

[cit19] Chen L., Shi G., Shen J., Peng B., Zhang B., Wang Y., Bian F., Wang J., Li D., Qian Z., Xu G., Liu G., Zeng J., Zhang L., Yang Y., Zhou G., Wu M., Jin W., Li J., Fang H. (2017). Nature.

[cit20] Wang Z., Huang L., Dong X., Wu T., Qing Q., Chen J., Lu Y., Xu C. (2023). Nat. Commun..

[cit21] Liang J., Zhang X., Liu T., Gao X., Liang W., Qi W., Qian L., Li Z., Chen X. (2022). Adv. Mater..

[cit22] Yang C., Long M., Ding C., Zhang R., Zhang S., Yuan J., Zhi K., Yin Z., Zheng Y., Liu Y., Wu H., Jiang Z. (2022). Nat. Commun..

[cit23] Liu T., Zhang X., Liang J., Liang W., Qi W., Tian L., Qian L., Li Z., Chen X. (2023). Nano Lett..

[cit24] Zhang W., Xu H., Xie F., Ma X., Niu B., Chen M., Zhang H., Zhang Y., Long D. (2022). Nat. Commun..

[cit25] Shen J., Liu G., Huang K., Chu Z., Jin W., Xu N. (2016). ACS Nano.

[cit26] Wu T., Wang Z., Lu Y., Liu S., Li H., Ye G., Chen J. (2021). Adv. Sci..

[cit27] Chu J., Huang Q., Dong Y., Yao Z., Wang J., Qin Z., Ning Z., Xie J., Tian W., Yao H., Bai J. (2022). Chem. Eng. J..

[cit28] Wang Z., Huang L., Dong X., Wu T., Qing Q., Chen J., Lu Y., Xu C. (2023). Nat. Commun..

[cit29] Wang Z., Hu H., Huang L., Lin F., Liu S., Wu T., Alharbi N. S., Rabah S. O., Lu Y., Wang X. (2020). Chem. Eng. J..

[cit30] Joshi R. K., Carbone P., Wang F. C., Kravets V. G., Su Y., Grigorieva I. V., Wu H. A., Geim A. K., Nair R. R. (2014). Science.

[cit31] Dikin D. A., Stankovich S., Zimney E. J., Piner R. D., Dommett G. H. B., Evmenenko G., Nguyen S. T., Ruoff R. S. (2007). Nature.

[cit32] Huang L., Guan T., Su H., Zhong Y., Cao F., Zhang Y., Xia X., Wang X., Bao N., Tu J. (2022). Angew. Chem., Int. Ed..

[cit33] Li T., Pickel A. D., Yao Y., Chen Y., Zeng Y., Lacey S. D., Li Y., Wang Y., Dai J., Wang Y., Yang B., Fuhrer M. S., Marconnet A., Dames C., Drew D. H., Hu L. (2018). Nat. Energy.

[cit34] Wan C., Liu Y., Feng P., Wang W., Zhu L., Liu Z., Shi Y., Wan Q. (2016). Adv. Mater..

[cit35] Liu H., Zhang X., Lv Z., Wei F., Liang Q., Qian L., Li Z., Chen X., Wu W. (2023). JACS Au.

[cit36] Breaker R. R., Joyce G. F. (1994). Chem. Biol..

[cit37] Lee J. H., Wang Z., Liu J., Lu Y. (2008). J. Am. Chem. Soc..

[cit38] Liu J., Brown A. K., Meng X., Cropek D. M., Istok J. D., Watson D. B., Lu Y. (2007). Proc. Natl. Acad. Sci. U. S. A..

[cit39] Nesterova I. V., Nesterov E. E. (2014). J. Am. Chem. Soc..

[cit40] Shi L., Peng P., Du Y., Li T. (2017). Nucleic Acids Res..

[cit41] Li L., Jiang Y., Cui C., Yang Y., Zhang P., Stewart K., Pan X., Li X., Yang L., Qiu L., Tan W. (2018). J. Am. Chem. Soc..

[cit42] Zhang X., Pan L., Guo R., Zhang Y., Li F., Li M., Li J., Shi J., Qu F., Zuo X., Mao X. (2022). Chem. Commun..

[cit43] Liu J. (2012). Phys. Chem. Chem. Phys..

[cit44] Brown A. K., Liu J., He Y., Lu Y. (2009). ChemBioChem.

[cit45] Cai W., Piner R. D., Stadermann F. J., Park S., Shaibat M. A., Ishii Y., Yang D., Velamakanni A., An S. J., Stoller M., An J., Chen D., Ruoff R. S. (2008). Science.

[cit46] Mkhoyan K. A., Contryman A. W., Silcox J., Stewart D. A., Eda G., Mattevi C., Miller S., Chhowalla M. (2009). Nano Lett..

[cit47] Szabó T., Berkesi O., Forgó P., Josepovits K., Sanakis Y., Petridis D., Dékány I. (2006). Chem. Mater..

[cit48] Yuan Y., Zhao S., Wen J., Wang D., Guo X., Xu L., Wang X., Wang N. (2019). Adv. Funct. Mater..

[cit49] Chen M., Liu T., Zhang X., Zhang R., Tang S., Yuan Y., Xie Z., Liu Y., Wang H., Fedorovich V. K., Wang N. (2021). Adv. Funct. Mater..

[cit50] Yan B., Ma C., Gao J., Yuan Y., Wang N. (2020). Adv. Mater..

[cit51] Wang D., Song J., Wen J., Yuan Y., Liu Z., Lin S., Wang H., Zhao S., Zhao X., Fang M., Lei M., Li B., Wang N., Wang X., Wu H. (2018). Adv. Energy Mater..

[cit52] Yuan Y., Niu B., Yu Q., Guo X., Guo Z., Wen J., Liu T., Zhang H., Wang N. (2020). Angew. Chem., Int. Ed..

[cit53] Yu Q., Yuan Y., Feng L., Feng T., Sun W., Wang N. (2020). Angew. Chem., Int. Ed..

[cit54] Gao J., Yuan Y., Yu Q., Yan B., Qian Y., Wen J., Ma C., Jiang S., Wang X., Wang N. (2016). Chem. Commun..

[cit55] Yuan Y., Feng S., Feng L., Yu Q., Liu T., Wang N. (2020). Angew. Chem..

[cit56] Yuan Y., Yu Q., Cao M., Feng L., Feng S., Liu T., Feng T., Yan B., Guo Z., Wang N. (2021). Nat. Sustain..

[cit57] Liu T., Gu A., Wei T., Chen M., Guo X., Tang S., Yuan Y., Wang N. (2023). Small.

[cit58] Chen M., Liu T., Tang S., Wei T., Gu A., Zhang R., Liu Y., Wang H., Xie Z., Yuan Y., Li Z., Wang N. (2022). Chem. Eng. J..

[cit59] Marvaniya K., Maurya A., Dobariya P., Kaushik A., Prakash P., Bhargava J., Patel K., Kushwaha S. (2022). Eur. Polym. J..

[cit60] Maurya A., Marvaniya K., Dobariya P., Mane M. V., Tothadi S., Patel K., Kushwaha S. (2024). Small.

[cit61] Feng L., Wang H., Feng T., Yan B., Yu Q., Zhang J., Guo Z., Yuan Y., Ma C., Liu T., Wang N. (2022). Angew. Chem., Int. Ed..

[cit62] Bao Q., Zhang D., Qi P. (2011). J. Colloid Interface Sci..

