# Supplementary material for: Designing biomimetic two-dimensional channels for uranium separation from seawater
Source: Chem Sci. 2024 Jun 10;15(27):10455–63. doi: 10.1039/d4sc02801e (PMC11234859; doi:10.1039/d4sc02801e)
Supplement: SC-015-D4SC02801E-s001 [file SC-015-D4SC02801E-s001.pdf]

Electronic Supplementary Material (ESI) for Chemical Science.
This journal is © The Royal Society of Chemistry 2024

Supporting information for

Designing Biomimetic Two-Dimensional Channels for Uranium Separation from Seawater

Wenbin Liang,[a,b] Xin Zhang,[a,b] Liqin Wang,[a,b] Chuanxi Wen,[a,b] Longlong Tian,[a,b,*] Zhan Li,[a,b,c,*] Ximeng Chen,[a,b] Wangsuo Wu,[a,b]

[a] MOE Frontiers Science Center for Rare Isotopes, Lanzhou University, Lanzhou 730000, China

[b] School of Nuclear Science and Technology, Lanzhou University, Lanzhou 730000, China

[c] School of Chemistry and Chemical Engineering, Qinghai Minzu University, Xining 810007, China

1. Experimental

1.1 Materials.

Graphite powder (200 mesh, 99%) , Sodium nitrate ($NaNO_3$, AR) , Concentrated sulfuric acid (H_2SO_4, 98%, AR) , Potassium permanganate ($KMnO_4$, GR) , Hydrogen peroxide (H_2O_2, 30%, AR) , Concentrated hydrochloric acid (HCl, 36%, AR) , Uranyl nitrate hexahydrate ($UO_2(NO_3)_2{\cdot}6H_2O$, AR) , Sodium chloride (NaCl, AR) , Potassium chloride (KCl, AR) , Calcium chloride ($CaCl_2$, AR) , Magnesium chloride hexahydrate ($MgCl_2{\cdot}6H_2O$, AR) , Copper nitrate trihydrate ($Cu(NO_3)_2{\cdot}3H_2O$, AR) , Zinc chloride ($ZnCl_2$, AR) and Nickel nitrate hexahydrate ($Ni(NO_3)_2{\cdot}6H_2O$, AR) were purchased from aladdin.

1.2 Preparation of Graphene Oxide

Graphene oxide (GO) prepared by modified Hummer method. First, add 5 g of 200 mesh graphite powder and 5 g of $NaNO_3$ into a 1L three-necked flask, slowly add 200 mL of concentrated sulfuric acid, and stir in an ice bath at 200 rpm for 1 h. Then, slowly add 20 g of $KMnO_4$, and react in a 38 ℃ water bath for 26 h. Then increase the stirring speed to 300 rpm and slowly add 250 mL of deionized water. Then transferred to a hot water bath at 84 ℃ for 15 minutes. Then the reaction solution was poured into a large beaker containing 500 mL of deionized water, and 30% H_2O_2 was added dropwise while stirring until there were no bubbles. After standing, the layers were separated, and the supernatant was discarded. Wash 3 times by centrifugation with 10% HCl. Finally, it was washed with deionized water until neutral.

1.3 Adsorption experiment

GO adsorption to DNA: Prepare a suspension containing GO at a concentration of 0.5 g/L and DNA at 10 μmol/L. Shake on a constant temperature shaker for 30 minutes. Subsequently, filter the homogenized solution through a 0.22 μm syringe filter. Finally, analyze the filtrate using a UV-visible spectrophotometer set at a wavelength of 260 nm to assess the DNA concentration accurately.

Adsorption selectivity of DNA for uranyl ions: Prepare a simulated seawater solution following the concentrations outlined in Table S1. Take 20 mL of the prepared simulated seawater and introduce 0.2 μmol of DNA. Agitate the solution on a thermostatic shaker for 30 minutes. Subsequently, precipitate the DNA molecules by adding ethyl acetate, followed by removal of the precipitate through centrifugation. Utilize an inductively coupled plasma optical emission spectrometer (ICP-OES) to analyze the concentration of each ion present in the solution. The selectivity of ions was assessed by calculating the *SF* for each ion, in which

$$SF = \frac{U_{\mathrm{ads}} / U_{\mathrm{aq}}}{C_{\mathrm{ads}} / C_{\mathrm{aq}}} \qquad (1)$$

where U_{ads} (mg g^{-1}) is the concentration of uranium adsorbed in the adsorbent, U_{aq} (mg g^{-1}) is the concentration in the spiked seawater, C_{ads} (mg g^{-1}) is the concentration of coexisting ions in the adsorbent, and C_{aq} (mg g^{-1}) is the concentration of coexisting ions in the spiked simulated seawater.

1.4 Preparation of GO-DNA membrane

To prepare GO-DNA membranes, a suspension of GO (0.5 g/L) and DNA (1 μM, 2 μM and 3 μM) is first prepared and stirred for 30 minutes to allow the DNA molecules to adsorb onto the surface of the GO nanosheets. The mixed suspension is then filtered under negative pressure on a polyethersulfone ultrafiltration membrane substrate with a specification of 0.22 μm and a diameter of 50 mm. The solvent is removed during the filtration process, and the GO nanosheets stack together to form the GO-DNA membrane. The π-π interaction between the GO nanosheets is a key factor in the formation of the GO-DNA membrane. This interaction occurs between the aromatic rings of the GO nanosheets, and it promotes the stacking of the nanosheets into a layered structure. Adsorption of DNA molecules to the surface of GO nanosheets also contributes to membrane formation. All bases in DNA are aromatic and can bind to GO through π-π stacking. This interaction can enhance the stability of the GO-DNA membrane by promoting the stacking of the GO nanosheets and strengthening the interactions between the DNA

molecules and the GO surface.

1.5 Transmission Electron Microscopy Characterization Method of GO-DNA Membrane

In order to prepare GO-DNA suspension for further analysis, the membranes were first mechanically exfoliated from polyethersulfone ultrafiltration membrane substrates and placed in a centrifuge tube along with an appropriate amount of deionized water. The mixture was then sonicated to obtain a homogeneous GO-DNA suspension. An aliquot of the suspension was dropped onto a micro-grid carbon support membrane purchased from Beijing Zhongjingkeyi Technology Co., Ltd, and dried under nitrogen gas. The resulting sample was then analyzed for morphology using a Tecnai F30 Transmission Electron Microscope in the Netherlands.

1.6 Atomic Force Microscopy Characterization Method of GO-DNA Membrane

To prepare the GO-DNA suspension, the method described in 1.5 was followed. An appropriate amount of the resulting suspension was then added dropwise onto a single crystal silicon wafer that had been treated using the RCA method. The sample was then analyzed for morphology using a Bruker Dimension Icon in Germany.

1.7 Scanning Electron Microscopy Characterization Method of GO-DNA Membrane

The GO-DNA membrane, obtained by mechanically peeling it off from the polyethersulfone ultrafiltration membrane substrate, was subjected to further analysis. The membrane was frozen in liquid nitrogen for 5 minutes and then broken to reveal its cross-section. The resulting cross-section was mounted on a sample stage and analyzed for morphology using an Apreo S scanning electron microscope (SEM) in the USA.

1.8 Separation experiment

Permeable separation experiment: The first step was to clamp the GO-DNA separation membrane into the separation device as shown in Figure S1a. Once the

membrane was in place, adjust the manual fastening table to fix it firmly. Next, the simulated seawater solution specified in Supporting Table 1 was prepared. The acidity of the mixed ion solution was adjusted with hydrochloric acid, and injected into the source solution tank located on the left side of the separation device. Prepare hydrochloric acid drive solutions with different acidities and inject them into the drive solution tank on the right side of the separation device. Stir the solutions at a constant temperature of 25 ℃ for 12 hours. Finally, measure the ion concentrations using an ICP-OES to determine the effectiveness of the separation. The instrument used is Agilent ICP-OES 725 from the United States. During the experiment, the concentration of uranium was lower than the detection limit of the Agilent ICP-OES 725. At this time, a trace uranium analyzer (WGJ-III) of Hangzhou Daji Optoelectronic Instrument Co., Ltd., China, was used for further analysis.

Pressure filtration experiment: First place the GO-DNA membrane on the support network. Install sealing gasket. Add the solution to be separated into the source solution tank. The sealed experimental device is connected to compressed air with a pressure of 0.08Mpa. Collect the filtrate. Finally, measure the ion concentrations using an ICP-OES to determine the effectiveness of the separation.

1.9 Separation device

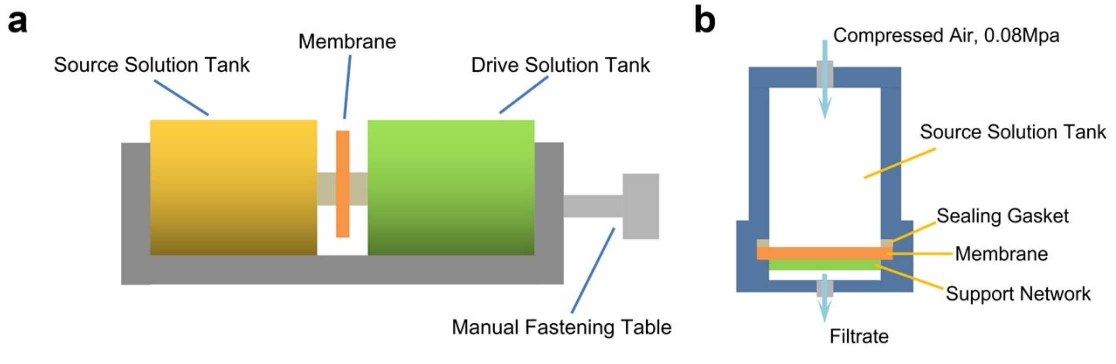

Figure S1. Separation device. (a) Custom-made permeable membrane separation device. (b) Pressure filtration experimental device

1.10 Separation result calculation

The separation effect of GO-DNA was evaluated by the transmittance, rejection

rate and separation factor according to the ion concentration measured by ICP-OES. The transmittance is calculated using Equation 2.

$$T = \frac{C_1}{C_0} \times 100\% \qquad (2)$$

Where T represents the transmittance (%), C_1 represents the ion concentration (M) after separation, and C_0 represents the ion concentration (M) before separation.

The rejection rate is calculated using Equation 3.

$$R = \frac{C_0 - C_1}{C_0} \times 100\% \qquad (3)$$

Among them, R represents the rejection rate (%), C_1 represents the ion concentration (M) in the filtrate, and C_0 represents the ion concentration (M) of the original solution.

The separation factors was calculated with Equation 4, according to the ratio of transmittance and rejection rate.

$$SF = \frac{T_M}{T_U}\left(T_M \geq T_U\right)$$
$$SF = \frac{T_U}{T_M}\left(T_U \geq T_M\right) \qquad (4)$$

Where SF represents the separation factor, T_M represents the transmission or the rejection rate of M ions (M=Na^+, K^+, Ca^{2+}, Mg^{2+}, Cu^{2+}, Zn^{2+}, Ni^{2+}), and T_U represents the transmission or the rejection rate of uranyl ions.

1.11 Concentration of Spiked Simulated Seawater

Supplementary Table S1. Concentrations of ions in real seawater and spiked simulated seawater

Element	Ions	Real sea water（ppb）	Spiked simulated seawater（ppb）
Na	Na^+	10.26×10^6	10.26×10^6
K	K^+	0.65×10^6	0.65×10^6
Ca	Ca^{2+}	0.92×10^6	0.92×10^6
Mg	Mg^{2+}	1.22×10^6	1.22×10^6

Cu	Cu^{2+}	2.8	2.8×10^3
Ni	Ni^{2+}	0.9	0.9×10^3
Zn	Zn^{2+}	1.05	1.05×10^3
V	VO^{2+}	2.6	2.6×10^3
U	UO_2^{2+}	3.3	3.3×10^3

1.12 Antibacterial experiments methods

Antibacterial experiments were conducted using Escherichia coli to evaluate the antibacterial efficacy of GO-DNA membranes. The culture medium comprising yeast extract (0.5% wt%), peptone (1% wt%), sodium chloride (1% wt%), and deionized water (97.7% wt%) was prepared. Escherichia coli was cultured in this medium to promote proliferation. The GO-DNA membrane under test was subjected to UV irradiation on both sides for 1 hour each to eliminate surface bacteria. Proliferated Escherichia coli bacterial liquid was then dropwise applied onto the surfaces of both the GO membrane and the GO-DNA membrane, followed by covering with a glass slide and gentle pressing for even dispersion. A control group received only bacterial solution. Incubation at 37°C for 2 hours allowed bacterial interaction. Subsequently, the membrane surfaces were rinsed with physiological saline, and the rinse fluid was collected and spread on solid culture medium, which was then incubated at 37°C for 24 hours. The number of bacterial colonies on the solid medium was counted to assess the antibacterial performance of the GO-DNA membrane, following standard protocols for antibacterial testing.

2. DNA Sequence

The sequence of the DNA chain is shown below (green represents the poly $A_{(10)}$ fragment, blue represents the i-motif fragment, and red represents the aptamer fragment that selectively recognizes uranyl ions), purchased from Suzhou Biosyntech Co., Ltd.

5'-AAAAAAAAAACCCCCTTTTCCCCCATCTCTGCAGTCGGGTAGTTAAACCGACCTTCAGACAGAGAACCCCCTTTTCCCCC-3'

3. Supplementary Table

Supplementary Table S2. The number of colonies of different types of membranes in antibacterial experiments

Membrane type	Number of colonies
Blank	~ 2300
GO	47
GO-DNA	25

Supplementary Table S3. U/V separation performance comparison.

Separate materials	U/V separation factor	References
SMON–PAO	1.79	[48]
BP@CNF-MOF	13.54	[49]
Zn^{2+}–PAO	1.3	[50]
PIDO NF	1.61	[51]
SSUP FIBER	6.17	[7]
BP-PAO Fiber	1.19	[52]
DSUP Fiber	8.73	[53]
CP-PAO	2.7	[54]
UiO-66-C_3N_4	11.42	[55]
PPH	2.37	[56]
Cu SA@UiO-66-NH_2	9.16	[57]
TCPP（Ni）-MOF-3	4.97	[58]
*p*TF	2.83	[59]
^{CSMCRI}HOF2-P	2.53	[60]
UO_2-Imprinted Nanocage	14.7	[61]
DNA-UEH	18.94	[4]
GO-DNA	14.66	This work

4. Supplementary Figures

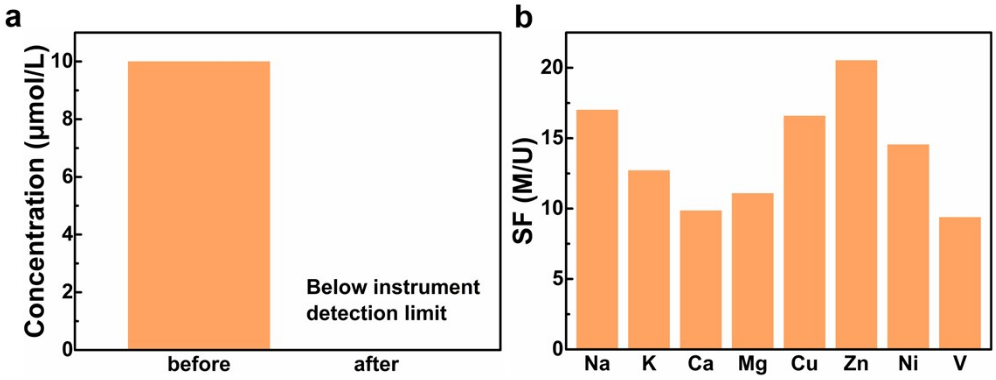

Figure S2. (a) Adsorption performance of designed DNA on GO. (b) Adsorption selectivity of the designed DNA in spiked simulated seawater.

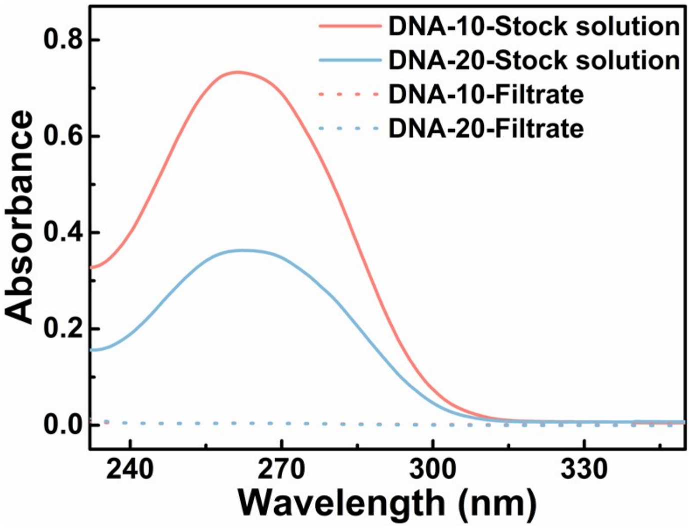

Figure S3. UV absorption spectra of stock solution and filtrate during membrane preparation.

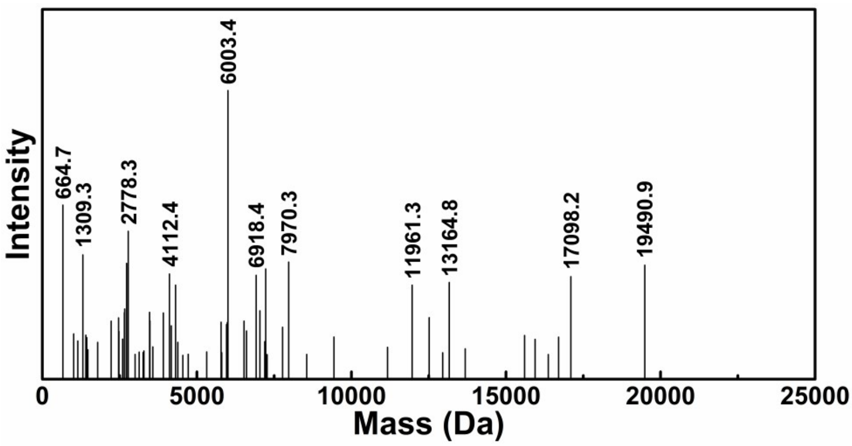

Figure S4. Mass spectrometry results indicate that DNA molecules are hydrolyzed at pH = 2.

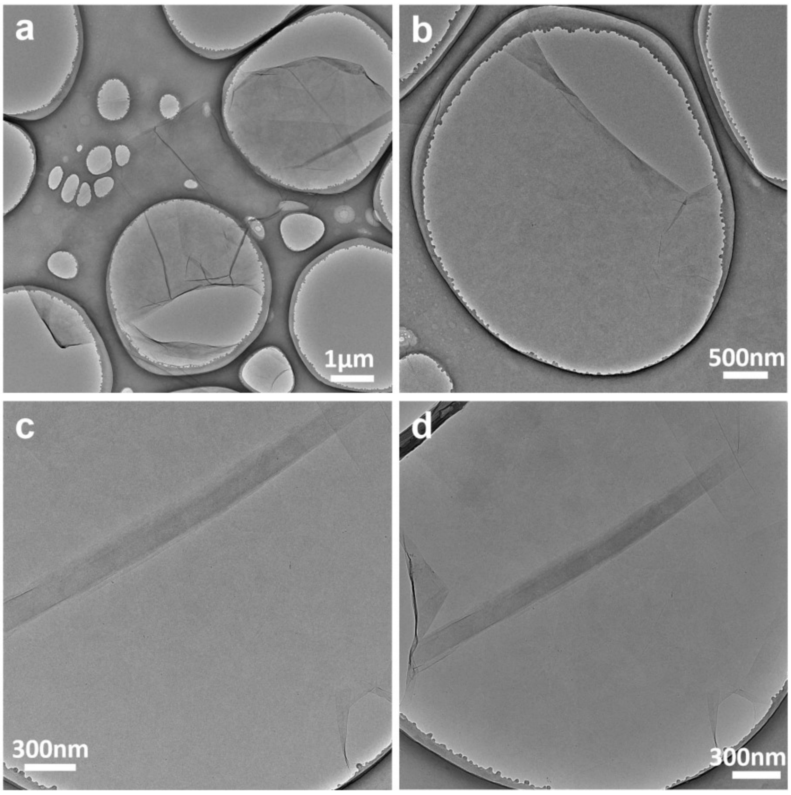

Figure S5. TEM image of GO.

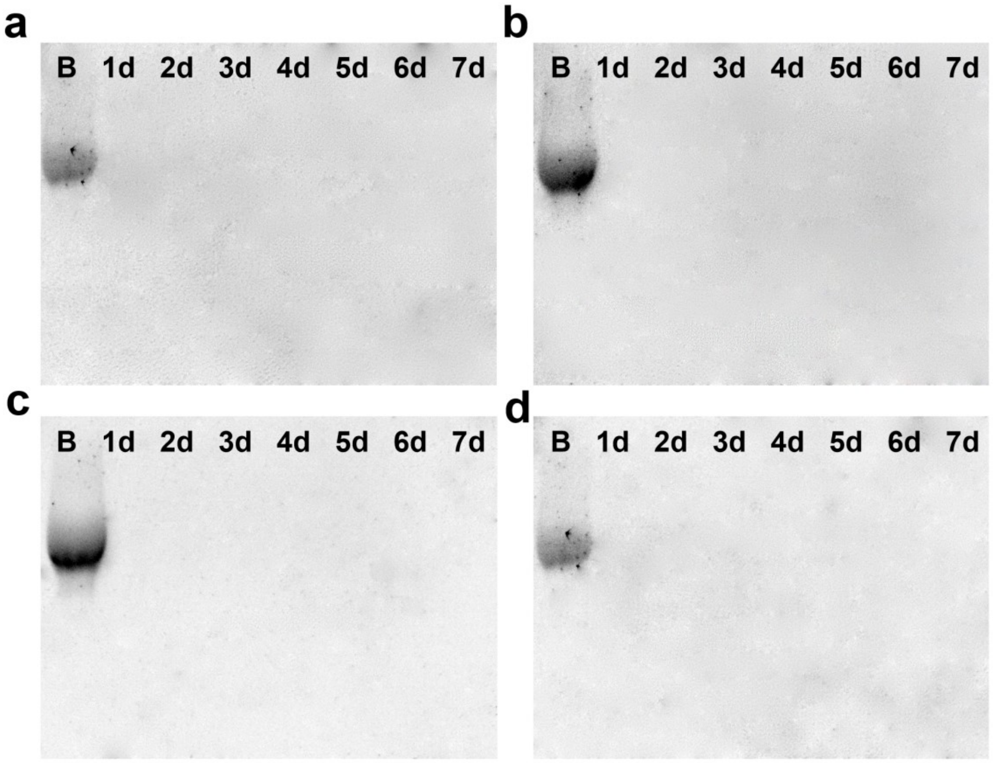

Figure S6. Gel electrophoresis analysis results of the obtained solutions at different pH (a is pH=2, b is pH=4, c is pH=6, d is pH=8) .

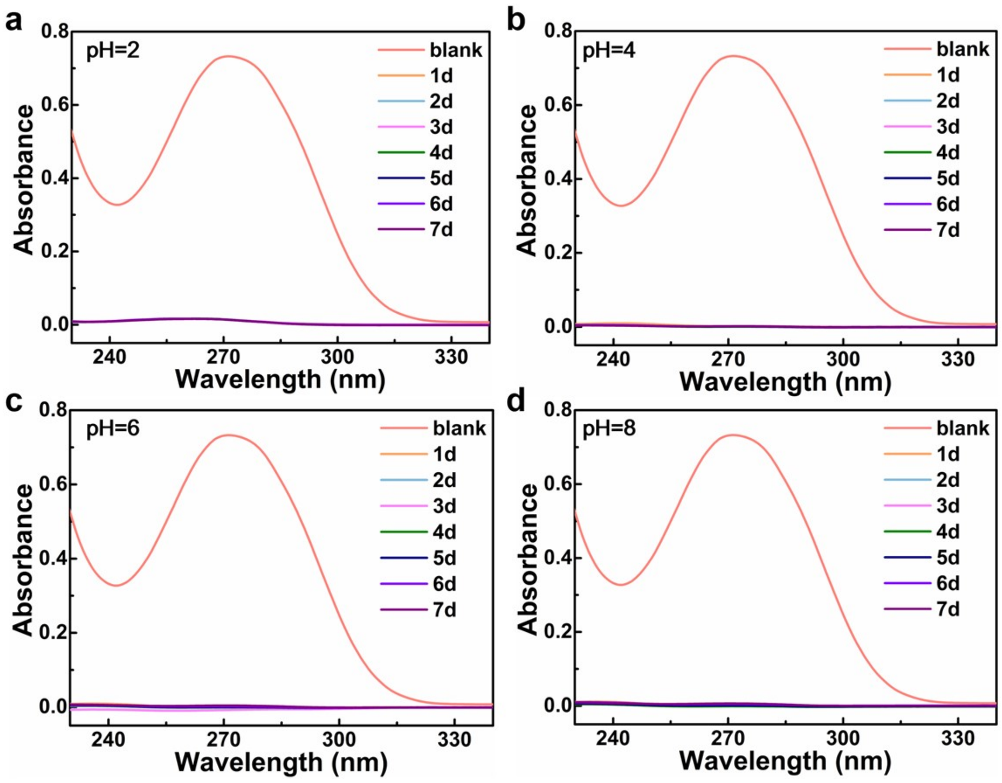

Figure S7. UV analysis results of GO-DNA membranes soaked in solutions with pH=2, 4, 6 and 8 for different times.

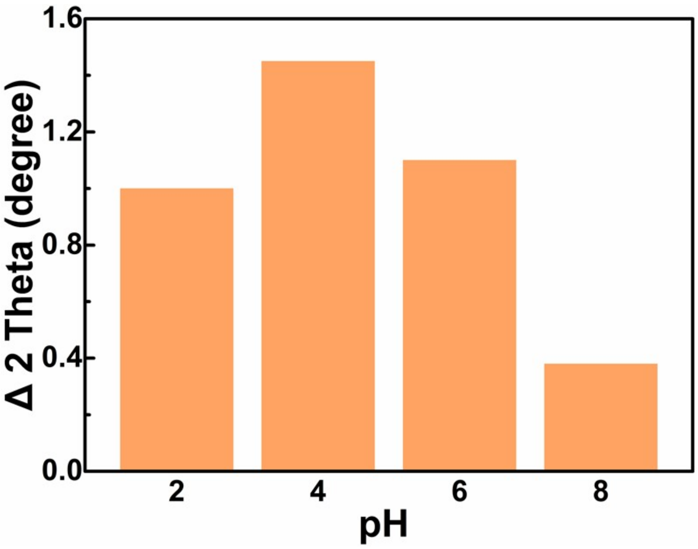

Figure S8. Left shift of 2 Theta degree of GO-DNA membrane compared to GO membrane at pH = 2, 4, 6 and 8

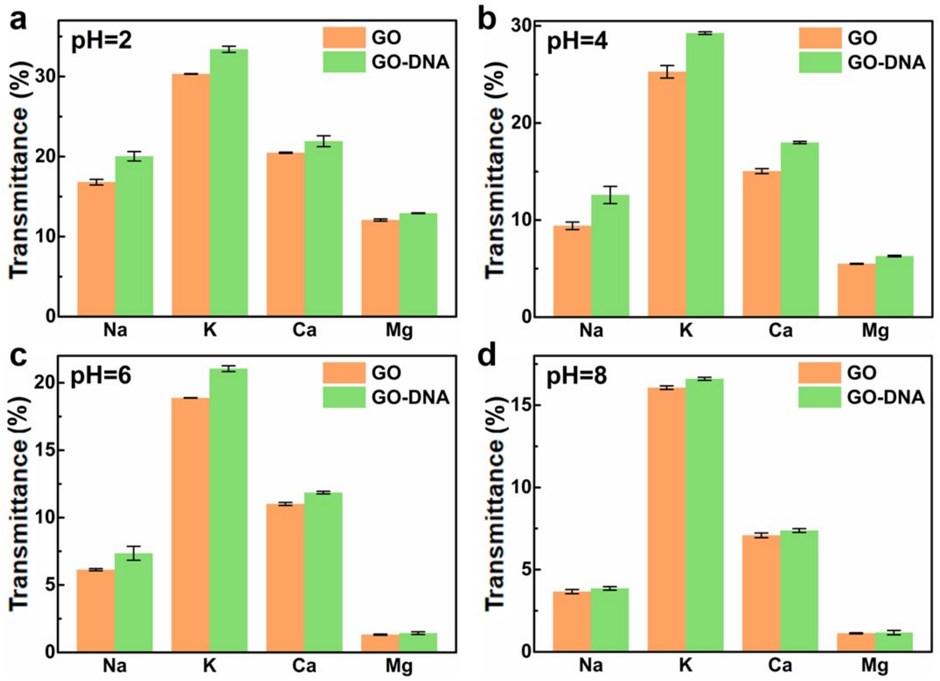

Figure S9. ionic transmittance of GO membrane and GO-DNA membrane at pH = 2, 4, 6 and 8

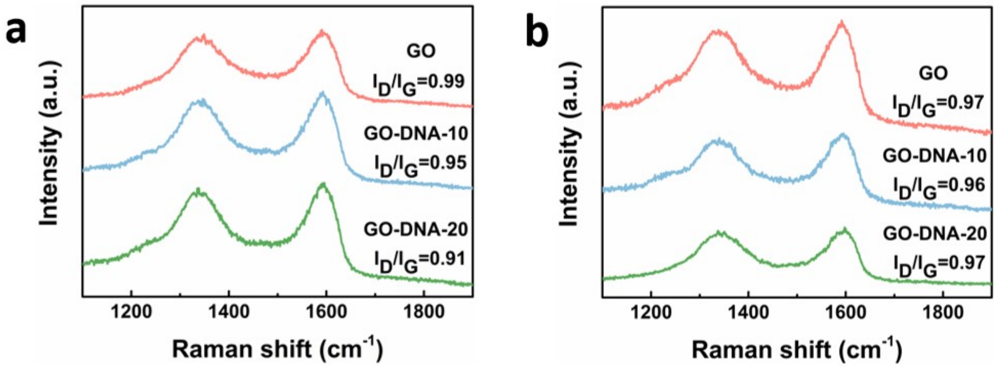

Figure S10. Raman spectra of GO membranes and GO-DNA membranes with different additions before (a) and after (b) separation.

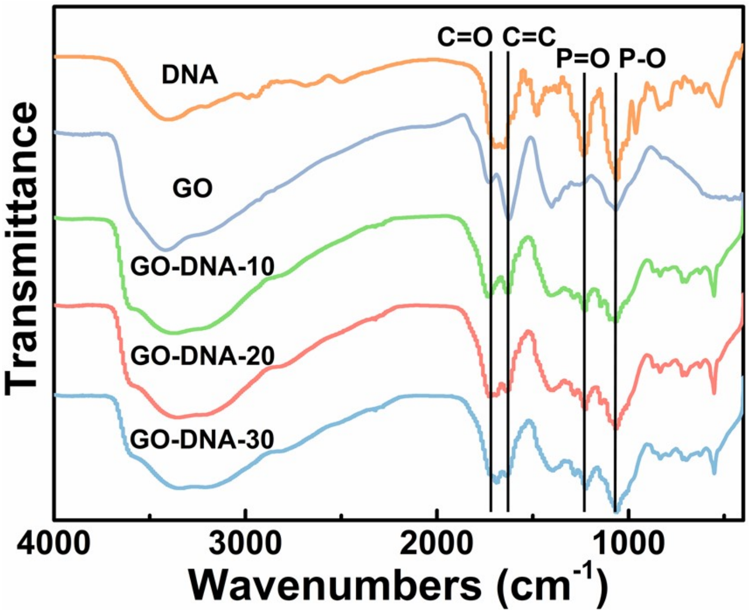

Figure S11. Fourier transform infrared spectroscopy (FT-IR) of GO membranes and GO-DNA membranes.

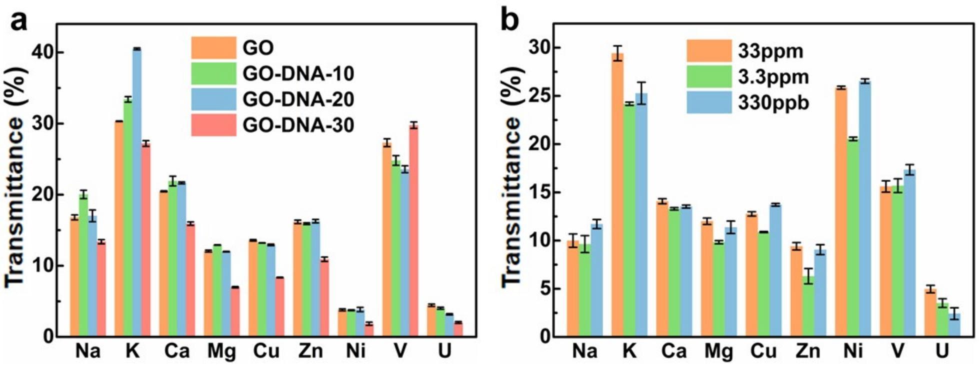

Figure S12. Transmittance after 12 hours of GO-DNA membrane separation experiment with different amounts of DNA added (a) and different uranium spiked concentrations (b).

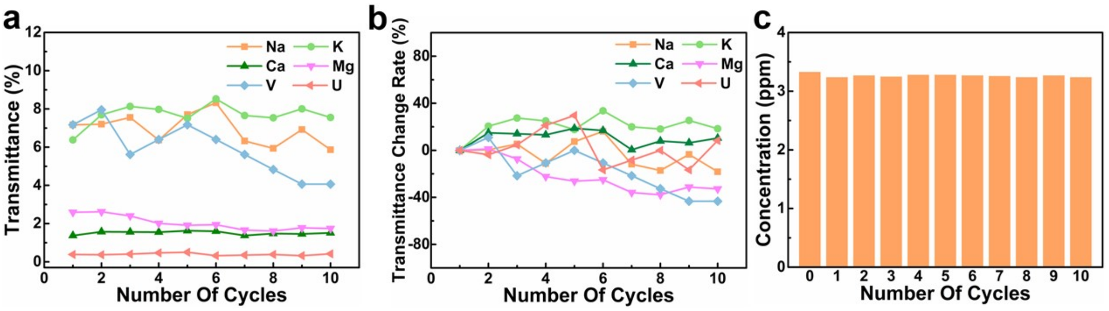

Figure S13. Transmittance (a) and Transmittance Change Rate (b) of GO-DNA membrane after ten cycles. (c) Concentration of uranyl ions in the source solution, 0 represents the uranyl ions concentration in the spiked real seawater before separation.

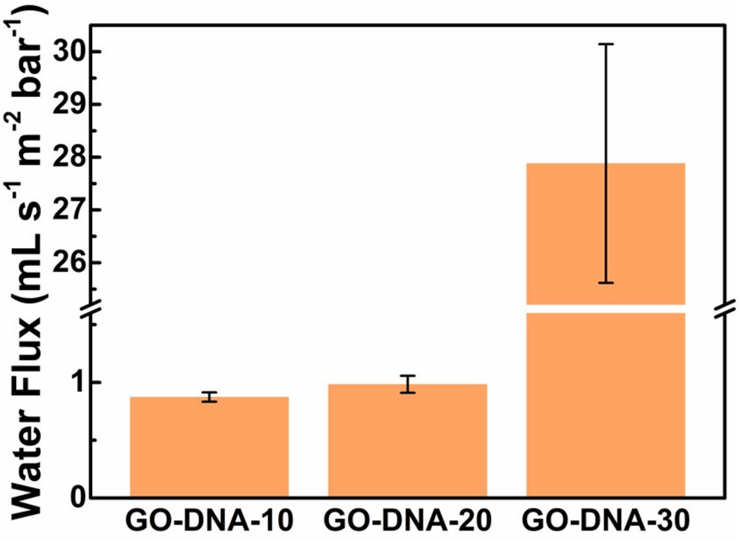

Figure S14. Water flux of GO-DNA membrane with different amounts of DNA added.

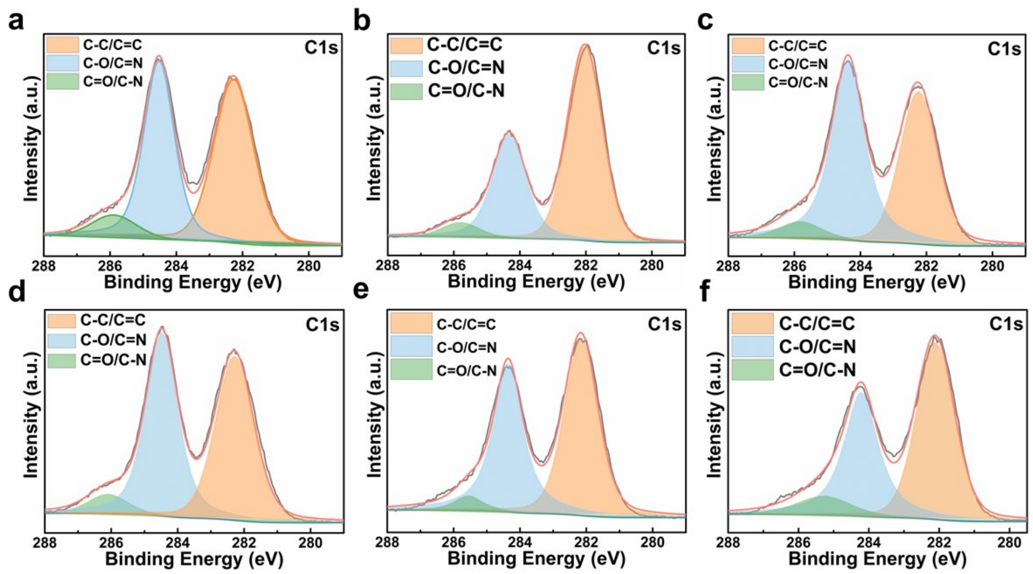

Figure S15. C-element high-resolution X-ray photoelectron spectroscopy (XPS) characterization results for GO membrane (a is before separation experiment, d is after separation experiment), GO-DNA-10 membrane (b is before separation experiment, e is after separation experiment) and GO-DNA-20 membrane (c is before separation experiment, f is after separation experiment).

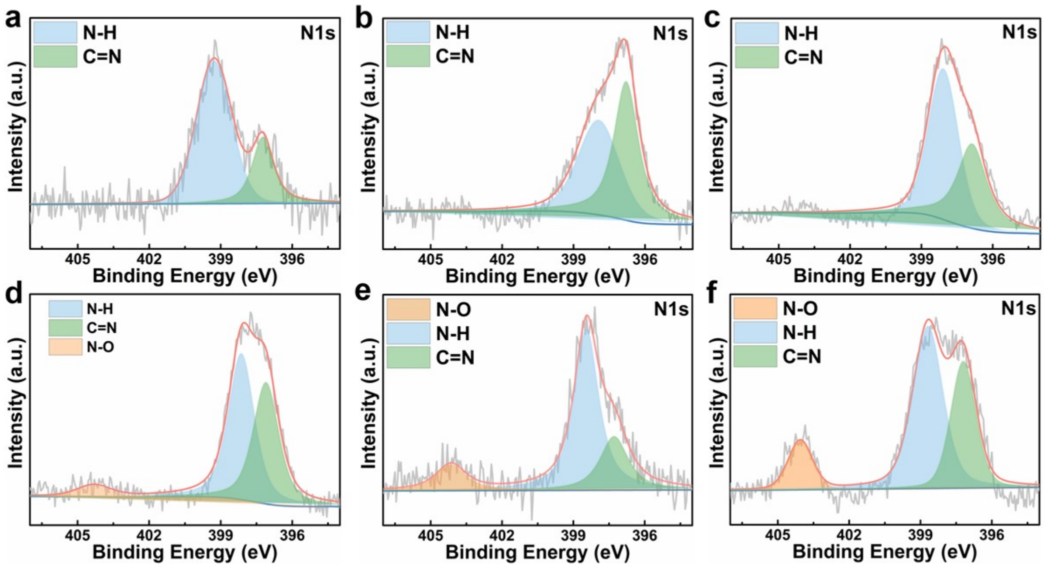

Figure S16. N-element high-resolution XPS characterization results for GO membrane (a is before separation experiment, d is after separation experiment), GO-DNA-10 membrane (b is before separation experiment, e is after separation experiment) and GO-DNA-20 membrane (c is before separation experiment, f is after separation experiment).

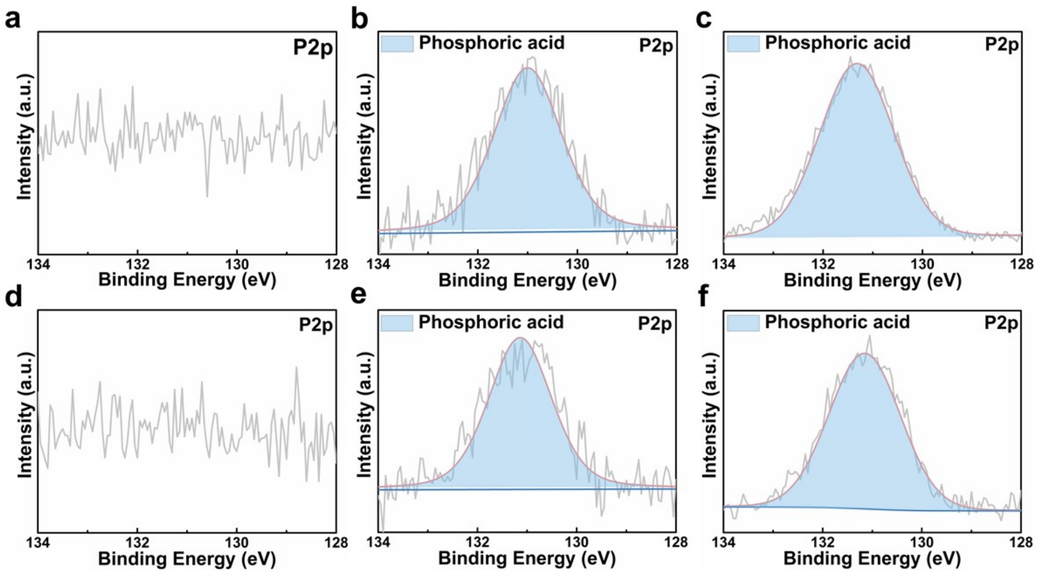

Figure S17. P-element high-resolution XPS characterization results for GO membrane (a is before separation experiment, d is after separation experiment), GO-DNA-10 membrane (b is before separation experiment, e is after separation experiment) and GO-DNA-20 membrane (c is before separation experiment, f is after separation experiment).

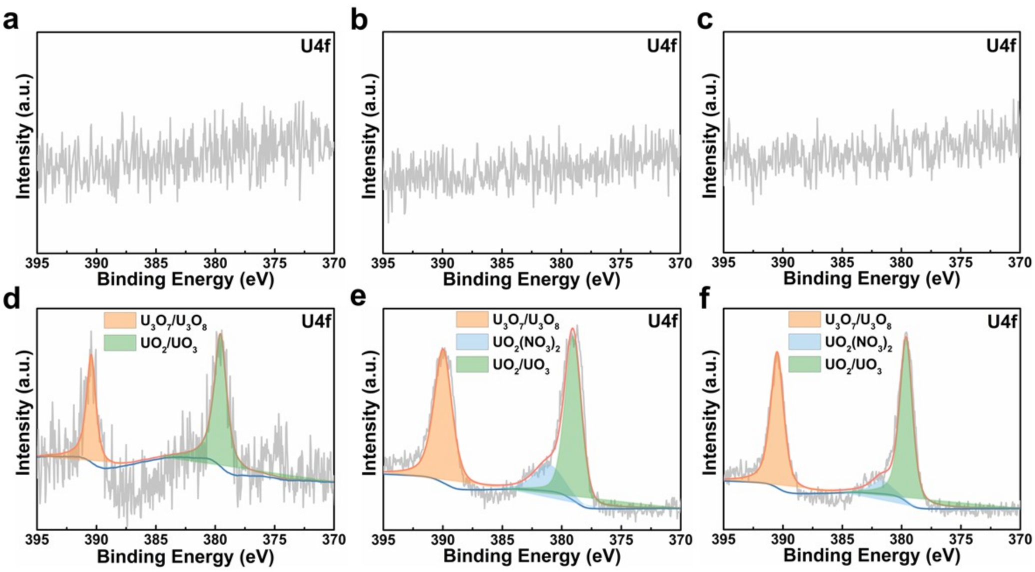

Figure S18. U-element high-resolution XPS characterization results for GO membrane (a is before separation experiment, d is after separation experiment), GO-DNA-10 membrane (b is before separation experiment, e is after separation experiment) and GO-DNA-20 membrane (c is before separation experiment, f is after separation experiment).
